# Metasurface-integrated atomic magnetometer using single-frequency dual beams

**DOI:** 10.1515/nanoph-2025-0295

**Published:** 2025-09-04

**Authors:** Ruofan Li, Shuo Sun, Jiahao Zhang, Jin Li

**Affiliations:** School of Instrumentation and Optoelectronic Engineering, Beihang University, Beijing, China; College of Optical and Electronic Technology, China Jiliang University, Hangzhou, China; National Institute of Extremely-Weak Magnetic Field Infrastructure, Hangzhou, China

**Keywords:** metasurface, atomic magnetometer, polarization conversion, high-sensitivity, miniaturization

## Abstract

Quantum sensing is rapidly advancing, creating new opportunities in fields such as fundamental science and advanced manufacturing. The optically pumped atomic magnetometer (OPM), known for its high sensitivity and compact form factor, has become a promising candidate for a range of applications. However, traditional OPM systems relying on bulky optical components and phase accumulation often face limitations in modulation precision and miniaturization. In this study, we introduce a miniaturized OPM design that incorporates a polarized metasurface and uses a single laser source for both pumping and detection. The polarized metasurface enables the transformation of linearly polarized light with arbitrary polarization azimuths into circularly polarized light, allowing for subwavelength-scale optical field manipulation and integrated beam splitting. In the developed system, both the circularly polarized pump beam and the zeroth-order linearly polarized probe beam are generated from the same metasurface device, forming a dual-path magnetometer architecture. This design significantly reduces the optical system size while achieving a sensitivity of 4.91 pT/Hz^1/2^ in the 2–10 Hz frequency range, exceeding the performance of conventional dual-beam OPMs with comparable configurations. Experimental results demonstrate that the proposed OPM effectively utilizes the metasurface’s optical properties to balance high sensitivity and system miniaturization. This work offers a practical approach for the integrated design of compact quantum sensing systems.

## 1 Introduction

Quantum sensors leveraging quantum entanglement can achieve enhanced sensitivity and accuracy that surpass those of conventional instruments [1], [2], [3], [4]. These sensors have been widely applied in various domains, including magnetic field sensing [5], [6], [7], [8], time-frequency metrology [9], [10], and nanoscale spatial resolution imaging [11], [12], [13]. The optically pumped atomic magnetometer (OPM), a typical quantum sensor, exhibits advantages including high resolution, non-invasive measurement capabilities, and cost-effectiveness [14], [15], [16], [17], [18]. With applications spanning mineral exploration, wearable health monitoring, and planetary magnetic field detection, there is an increasing demand for OPMs with enhanced sensitivity and simplified integration [19], [20], [21], [22]. However, the physical structure and performance requirements of OPM have imposed limitations on further miniaturization. On the one hand, achieving both exceptional functionality and a compact design simultaneously remains a significant challenge. Compared to dual-beam OPMs, single-beam OPMs feature fewer optical components, reduced optical path complexity, and easier integration, but its sensitivity remains constrained compared to the latter [23], [24], [25]. Dual-beam OPMs are often considered a better solution for high-sensitivity magnetic field measurements, but their architectural complexity and inherent optical path volume limit the space for miniaturization of magnetometers [26], [27], [28]. On the other hand, the physical limitations of traditional optical devices restrict the integration of OPMs. Conventional optics in the system demand sufficient dimensions and mass to accumulate phases to obtain the desired phase shifts profile, typically leading to bulky designs with restricted adjustability. These constraints not only hinder the enhancement of OPM performance but also restrict their broader application [29], [30], [31], [32], [33]. Overall, new technological strategies are needed for the development of high sensitivity integrated OPMs.

Metasurfaces facilitate subwavelength-scale manipulation of the phase, amplitude, and polarization of light and demonstrate high compatibility in semiconductor manufacturing processes. This characteristic endows them with substantial potential for system integration and miniaturization [34], [35], [36], [37], [38]. The integration of metasurfaces with OPMs is emerging as a significant direction for the development of high-performance quantum sensing systems, as evidenced by many recent studies. A dual-beam OPM scheme for planar nano-fabrication is proposed, in which the optical paths of the parallel-incident pump beam and probe beam are deflected and intersected by a metasurface [34]. Research on metasurface-based single-beam magnetometers has primarily focused on the incident-side optical field modulation and the polarization beam splitting at the detection end [39], [40], [41], [42], [43], [44]. To the best of our knowledge, the incident field conversion strategy and energy efficiency enhancement in both dual-beam and single-beam magnetometer architectures have been highlighted as critical aspects in metasurface-based OPM studies. However, the empirical analysis of zeroth-order light utilization in the conversion process remains an open area of research. Further investigations are also necessary to leverage the use of metasurfaces for enhancing OPM performance and realizing compact optical path structures.

**Figure 1:**
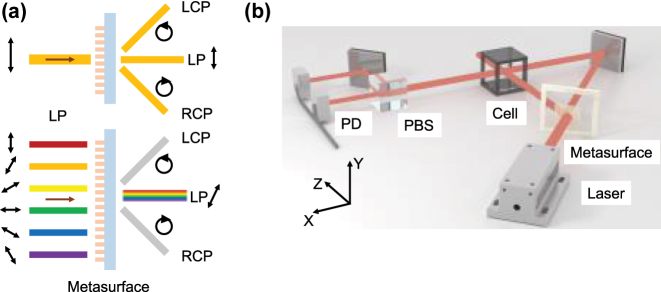
Designed metasurface-integrated atomic magnetometer using single-frequency dual beams. (a) Schematic diagram of metasurface functionality. LCP: left-handed circularly polarized light. RCP: right-handed circularly polarized light. (b) Conceptual diagram of the designed dual-beam OPM based on polarized metasurface. PBS: polarization beam splitter. PD: photodiode.

In this work, we propose and experimentally demonstrate a dual-beam OPM scheme based on a polarized metasurface and a single-frequency laser, which has a sensitivity of 4.91 pT/Hz^1/2^ within the 2–10 Hz frequency band. The designed and optimized metasurface is capable of converting linearly polarized light (LP) incident at any arbitrary polarization azimuth into circularly polarized light (CP) at the nanoscale, as shown in Figure 1a, while simultaneously enhancing the beam quality to a certain extent. The converted CP is angularly separated from the unconverted zeroth-order LP by a gradient phase, with the axis ratio (AR) of the converted CP maintained within 0.53 dB The previously unrecognized zeroth-order LP is utilized as the probe beam in the dual-beam OPM, thereby overcoming the conversion efficiency bottleneck caused by material properties and structural design constraints in prior studies. We employ the metasurface as the core for beam splitting, polarization transformation, and constructing a dual optical path system for individual lasers within a compact optical framework (Figure 1b). The atomic magnetometer system developed in this work achieves high sensitivity while maintaining a small footprint, combines the advantages of single-beam and dual-beam magnetometers, and presents a novel pathway for future performance enhancement and chip-scale integration of OPMs.

## 2 Design methods and result discussion

### 2.1 Principle

OPMs generally necessitate two beams that serve to induce magnetically sensitive state and measure the magnetic field, respectively. The former, a circularly polarized pump beam, transfers angular momentum to alkali metal vapor atoms to modify their quantum states. Through successive cycles of pumping, quenching, and relaxation, the atoms attain a polarized state. Once polarized, the disordered distribution of atomic magnetic moments evolves into an aligned configuration, thereby establishing a macroscopic net magnetic moment [45]. The interaction between magnetization vector *M* and external magnetic field *B* can be mathematically described using Bloch equation:

(1) $$\frac{\text{d}S}{dt}=\gamma S\times B+\frac{S_{0}-S}{T}$$

where *S* is the spin polarization vector, *γ* is the gyromagnetic ratio, *S*_0_ is the equilibrium magnetization vector, and *T* is the relaxation time. The probe beam conducts magnetic field measurements through polarimetry by detecting polarization rotation of the transmitted light. The polarization azimuth of the linearly polarized laser changes after traversing the highly polarized atomic vapor cell, and the transmitted laser light *I*_0_ is split into two beams using polarizing beam splitter (PBS) to measure the refracted and reflected light intensities I1 and I2:

(2) $$\left\{\begin{array}{c} I_{1}=I_{0}\text{co}{\text{s}}^{2}\left(\beta -\pi /4\right)\;\\ I_{2}=I_{0}\text{si}{\text{n}}^{2}\left(\beta -\pi /4\right)\; \end{array}\right.$$

*β* represents the optical rotation angle, which varies only slightly. Therefore, the following equation can be derived:

(3) $$I_{1}-I_{2}=I_{0}\sin\left(2\beta \right)\approx 2I_{0}\beta$$

Conventional optical components rely on bulk materials to manipulate light, and generally necessitate a certain inherent thickness to accumulate the required phase for effective control, typically featuring curved surfaces. This property impedes efforts to reduce the overall system volume. In contrast, metasurfaces could achieve phase modulation in an ultra-thin, and planar configuration through precisely designing the shape, size and spatial distribution of nanoscale unit cells [46]. Therefore, we have decided to replace traditional components in the magnetometer system with metasurfaces, as they are more conducive to miniaturization. When a plane wave propagates along the positive *z*-axis onto the metasurface, the conversion relationship between the incident optical field *E*_*i*_, and the outgoing optical field *E*_0_ can be expressed as:

(4) $$E_{0}=T\times E_{i}$$

where *T* denotes the 2 × 2 normalized Jones matrix in the Cartesian coordinate system, which can be obtained from transforming the Jones matrix *T*_0_ in the anisotropic *u*–*v* coordinate system by the transformation matrix Ω_0_. Its general form is given by:

$$T=\Omega_{0}^{-1}T_{0}\Omega_{0}$$

(5) $$={\left[\begin{array}{cc} \cos\zeta & \sin\zeta \\ -\sin\zeta & \cos\zeta \end{array}\right]}^{-1}\left[\begin{array}{cc} t_{u} & 0\\ 0 & t_{v}e^{j\varphi } \end{array}\right]\left[\begin{array}{cc} \cos\zeta & \sin\zeta \\ -\sin\zeta & \cos\zeta \end{array}\right]$$

$$=\left[\begin{array}{cc} t_{u}\cos^{2}\zeta +t_{v}\text{si}{\text{n}}^{2}\zeta e^{j\varphi } & \left(t_{u}-t_{v}e^{j\varphi }\right)\sin\zeta \cos\zeta \\ \left(t_{u}-t_{v}e^{j\varphi }\right)\sin\zeta \cos\zeta & t_{u}\text{si}{\text{n}}^{2}\zeta +t_{v}\text{co}{\text{s}}^{2}\zeta e^{j\varphi } \end{array}\right]$$

*t*_*u*_ and *t*_*v*_ represent the electric field transmission amplitudes along the nanorods’ two orthogonal principal axes corresponding to the incident polarization. *φ* is denoted as the phase delay between the two electric field components, and *ζ* is the rotation angle of the metasurface coordinate system with respect to the Cartesian system. The designed metasurface is capable of converting LP of arbitrary polarization azimuth separately into left-handed circularly polarized light (LCP) and right-handed circularly polarized light (RCP). Therefore, the complex amplitudes *t*_*u*_ and *t*_*v*_ are set to 1, the phase delay for the co-polarized transmission coefficients for *x*- and *y*-linear polarizations is set to *φ* = *π*/2, and the angle between the fast axis and the principal axis is defined as *ζ* = *π*/4. For incident *x*-polarized light, *E*_*i*_ = [1; 0], the transmitted field *E*_0_ can be expressed as:

(6) $$E_{0}=T\times E_{i}=\frac{\sqrt{2}}{2}e^{j\frac{\pi }{4}}\left[\begin{array}{c} 1\\ -j \end{array}\right]$$

namely, LP is converted to LCP. Accordingly, when the phase delay *φ* is set to −*π*/2, right-handed circularly polarized light (RCP) can be obtained. After analyzing the optical field modulation based on polarization conversion theory, the following section provides a detailed elaboration on polarization beam splitting. The refraction part of the generalized Snell’s law is presented as follows:

(7) $$n_{t}\sin\theta_{t}-n_{i}\sin\theta_{i}=\frac{\lambda_{0}}{2\pi }\frac{\text{d}\Phi }{dx}$$

*θi*, *θt* represent the incident and refracted angles. *n*_*i*_, *n*_*t*_ are the refractive indices of the incident and refractive media. *λ*_0_ is the wavelength of light in a vacuum. dΦ corresponds to the difference in phase discontinuities between adjacent crossing points on the interface. d*x* represents spacing between the crossing points. The spatial structure and period distribution of the metasurface are rigorously designed to introduce the phase discontinuity gradient dΦ/d*x*, thereby generating the anomalous refraction phenomenon. Since the laser source used in the OPM closely approximates a normally incident collimated beam (*θ*_*i*_ = 0), the following result is obtained:

(8) $$\theta_{t}=\arcsin\left(\frac{\lambda_{0}}{2\pi n_{t}}\frac{\text{d}\Phi }{dx}\right)$$

As can be observed from Equation (8), given that the refractive index and wavelength are fixed, an appropriate dΦ/d*x* can be regulated to realize the desired refraction angle. Consequently, the CP generated by the nanostructures exits the surface at an oblique angle *θ*_*t*_. Due to challenges in achieving precise phase delays across all frequency bands, as well as non-ideal structures arising from current fabrication processes, a fraction of the input optical energy inevitably manifests as unconverted zeroth-order light. One advantage of the designed metasurface is that the obliquely refracted CP is spatially separated from the zeroth-order LP, which propagates perpendicularly along the incident direction, thereby ensuring the purity of the converted light – especially when compared with traditional optical instruments that struggle to effectively isolate the unwanted zeroth-order light. On the other hand, we have chosen to utilize the zeroth-order light, which is typically regarded as a constraint on optical instrumentation, to enable novel functionalities. The high-quality CP obtained from the metasurface is employed as the pump beam for the OPM, while the centrally transmitted zeroth-order LP serves as the probe beam.

### 2.2 Metasurface design and simulation

**Figure 2:**
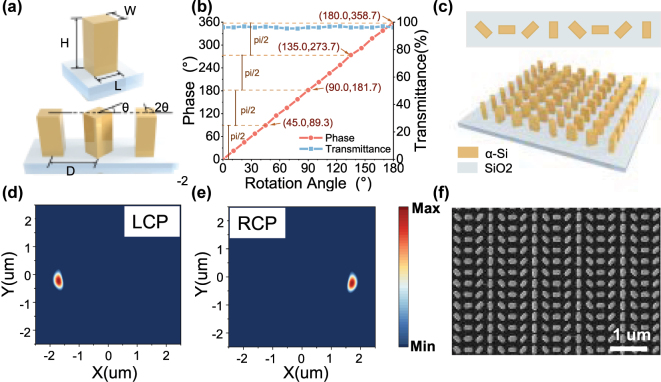
Design and Simulation of metasurface. (a) Structural diagram of metasurface nanopillar. (b) Simulated phase and transmittance of the outgoing light at each rotation angle. (c) Model diagram of polarized metasurface. (d–e) Electric vector distributions of LCP and RCP in the outgoing light field. (f) Metasurface SEM image.

Based on the aforementioned analysis, we designed and simulated the metasurface structure. We selected amorphous silicon (α-Si) as the nanopillar material and SiO_2_ as the substrate for the simulation experiments. The unit cell structure is depicted in Figure 2a. Through comprehensive parameter sweeps and optimization, the dimensions of the optimized transmittance near the D1 line of the ^87^Rb OPM were determined to be *L* = 220 nm, *W* = 110 nm, *H* = 600 nm (0.277*λ* × 0.138*λ* × 0.755*λ*), where *λ* is the operating wavelength of 795 nm, with a spacing *D* = 300 nm (0.377*λ*). A structural unit is formed by laterally arranging rectangular nanopillars, each rotated successively by a fixed angle increment *θ* until completing a full 360° cycle. The metasurface structure is implemented using this structural unit as the basic building block. For instance, when *θ* = *π*/3, six unit cells complete one full cycle. The phase differences and transmission coefficients corresponding to each rotation angle within the range of 0 to *π* were evaluated using the finite-difference time-domain (FDTD) software, and the results are presented in Figure 2b. To ensure a consistent phase discontinuity gradient across adjacent nanopillars, the spacing (d*x*) was fixed. As a result, a constant rotation angle increment (Δ*θ*) leads to a uniform phase shift (ΔΦ). By setting Δ*θ* = *π*/4, the phase values *φ* corresponding to *θ* = *π*/4, *π*/2, 3*π*/4, and *π* were determined. It is evident that, within the permitted precision, these four samples demonstrate a stable phase gradient with a phase error not exceeding 1.37 %. Other samples with varying initial orientations and Δ*θ* = *π*/4 can also meet this criterion. According to Equation (8), the converted LCP and RCP are refracted at an angle of 41.5°. The final array exhibits a rotational periodicity of *π*/4, where each structural unit comprises eight nanopillars arranged in a cyclic configuration, as shown in Figure 2c.

The polarization conversion performance of the structural unit design was assessed via simulations. LP is incident vertically, and the electric fields corresponding to the LCP and RCP in the *x*–*y* plane were separately extracted. As shown in Figure 2d, under the LCP component electric field, a distinct spot appears on the left side, while the electric vector intensity at the same position of the RCP component electric field (Figure 2e) remains below the detection threshold, indicating the absence of a light spot. This indicates that, from the perspective along the laser incident direction, the left beam corresponds to the converted LCP. Similarly, Figure 2e shows that a distinct spot appears on the right side under the RCP component electric field, whereas the LCP electric field component (Figure 2d) exhibits no detectable spot at that location, thereby confirming that the right beam corresponds to the converted RCP. Therefore, it can be conclusively demonstrated that the proposed metasurface design successfully enables the conversion of linearly polarized light to circularly polarized light (LTC), yielding high-purity LCP and RCP beams on the left and right sides, respectively. The microstructural morphology and geometrical characteristics of the metasurface fabricated using lithography and etching techniques were characterized by scanning electron microscopy (SEM), as depicted in Figure 2f. The image clearly illustrates the periodically arranged subwavelength structural units, which exhibit uniform shapes and sizes. Nanopillar dimensions generally span from the nanometer to the micrometer scale. However, due to limitations in fabrication precision, their edges are not sharply defined, thereby reducing the conversion efficiency of the metasurface and inducing partial zeroth-order light generation. Overall, the designed metasurface is capable of LTC conversion of incident laser light with arbitrary polarization azimuth.

### 2.3 Characterization of metasurface optical properties

**Figure 3:**
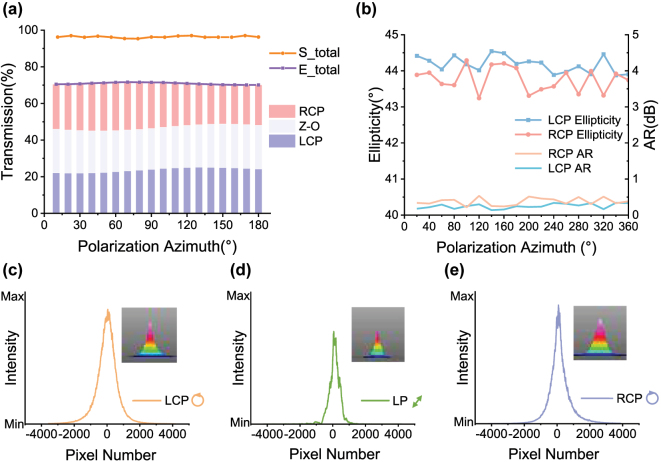
Characterization of metasurface optical properties. (a) Theoretical and experimental values of metasurface transmittance for incidence of LP at different polarization azimuths. S_total: simulated value of the total transmittance; E_total: experimental value of the total transmittance; Z–O: zeroth-order linearly polarized light; (b) polarization and axial ratio of the converted light for incidence of LP at different polarization azimuths. AR: axial ratio (c–e) intensity profiles and beam quality profiles of three outgoing beams on the metasurface.

The performance of the metasurface was assessed through a comprehensive series of experimental evaluation. The simulated and experimental transmittances of LPs with different polarization azimuths on the metasurface are presented in Figure 3a. At a wavelength of 795 nm, the simulated metasurface exhibits an average transmittance of 96.30 %, whereas the experimental measurement yields 70.84 %. As elaborated previously, this discrepancy may be attributed to fabrication inaccuracies, environmental influences during testing, and other potential factors. The measured intensities of the converted LCP and RCP components were nearly identical, thereby confirming the effectiveness of the metasurface design. Additionally, the intensity of the LP beam transmitted directly without modulation by the metasurface was found to be consistent with that of the LCP/RCP beams. This LP beam retains the same polarization angle as the incident light. In a dual-beam OPM, both the pump and probe beams are required to meet specific certain power thresholds. Moreover, the alkali vapor cell, when heated to the operating temperature, absorbs energy from the incident laser beam. If the disparity between the ratio of CP and LP induced by the metasurface is excessively large, the total power supplied to the OPM must be increased to ensure that the weaker branch meets the operational requirements, leading to unnecessary power consumption. Hence, generating pump and probe beams with comparable intensities through metasurface modulation offers a significant advantage for efficient system operation.

Define the axis ratio AR(dB) = 20∗log10(1/tan(*ɛ*)), where *ɛ* denotes the ellipticity. As AR approaches zero, the transmitted light tends toward ideal circular polarization. Figure 3b shows the ellipticity of LCP and RCP derived from LP with polarization conversion over a 0–360° azimuthal range. The average ellipticity of LCP is 44.18°, whose AR is only 0.14–0.34 dB; whereas the average ellipticity for RCP is 43.76° with an AR not exceeding 0.53 dB. Both of the beams meet the requirements for the dual-beam OPM pump beam. The polarization purity and stability of the zeroth-order linearly polarized light are validated in Supplementary Material S1. Since efficient polarization conversion requires a beam close to ideal circular polarization, we selected LCP – with the lower AR – as the pump beam in our OPM experiments. On the other hand, due to the symmetric micro-geometric characteristics of the designed metasurface, it can also enhance the beam quality of the incident plane wave to a certain extent while fulfilling the aforementioned functions. The intensity distributions along the beam centerline for the three transmitted beams – LCP, zeroth-order light, and RCP – are presented in Figure 3c–e, with insets showing the intensity distribution in the *x*–*z* plane. Experimental measurements of the beam quality of the two converted beams and the zeroth-order beam indicate that the output beams exhibit typical Gaussian profiles. High beam quality is beneficial for efficient optical pumping of the atoms and high-precision detection. Therefore, the experimental results validate that the metasurface operates in accordance with its design specifications, generating converted CP and zeroth-order transmitted LP with nearly equivalent intensities, while the converted CP exhibits superior polarization characteristics and beam quality. This is conducive to the advancement of a polarized metasurface-based OPM.

### 2.4 OPM system performance based on polarized metasurface

**Figure 4:**
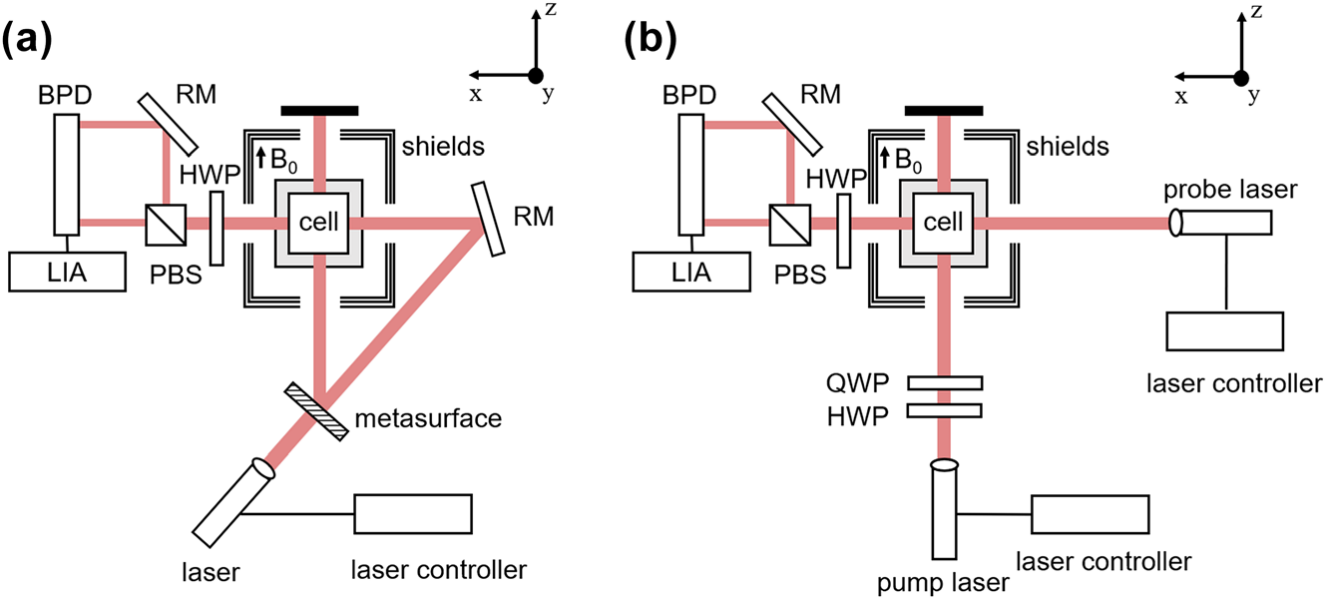
OPM system. (a) Structural diagram of OPM based on polarized metasurface. QWP: quarter-wave plate. HWP: half-wave plate. PBS: polarizing beam splitter. RM: reflecting mirror. BPD: balanced amplified photodiode. LIA: lock-in amplifier. (b) Structural diagram of dual-beam OPM based on conventional optics.

A metasurface-based OPM system was designed, constructed and experimentally evaluated. As shown in Figure 4a, the 795 nm linearly polarized laser emitted from the DFB laser first vertically traverses through the metasurface, where the entire polarization conversion is complete. Among the output beams, the LCP beam deviates to the left relative to the incident beam and is transmitted directly along the +*z* axis through the cell window, thereby functioning as the pump beam path. Meanwhile, the central zeroth-order beam, which does not undergo polarization conversion by the LTC metasurface, retains its initial linear polarization. After direction adjustment by a mirror, it functions as the probe beam, propagating perpendicularly to the pump beam along the +*x*-axis through the alkali-metal vapor cell. This trajectory is referred to as the probe beam path.

The SiO_2_ cell (10 × 10 × 10 mm^3^) contains ^87^Rb atoms and 700 Torr of N_2_ and is enclosed within a ceramic oven and a 20 × 20 × 20 mm^3^ PEEK cell frame. The cell frame, surrounded by three-axis Helmholtz radio frequency coils, was mechanically secured at the center of a five-layer permalloy shield, which mitigates the adverse effects of magnetic field inhomogeneity on the magnetic resonance linewidth. Experimental measurements reveal that the average remanent magnetic field within the shield is 1 nT. The coil inside the shield, characterized by a constant of 0.268 μT/mA, provides *z*-axis bias magnetic field *B*_0_. A HWP (MAHWP20-SNIR, LBTEK) positioned after the vapor cell is utilized during the polarization detection stage to balance the *p*- and *s*-polarized components separated by the PBS (PBS25-1, JCOPTIX). The differential intensity signal is directed into a lock-in amplifier, where the magnetic resonance amplitude and phase signals are demodulated. Additionally, we constructed an OPM based on conventional optical components, with a schematic diagram shown in Figure 4b. The traditional dual-beam OPM incorporates an additional set of lasers and associated control systems; the pump beam is sequentially transmitted through a HWP and a QWP (MAQWP20-SNIR, LBTEK) to generate circular polarization. In comparison with the metasurface-based OPM, the conventional configuration exhibits higher costs, requires more space, and demands more precise mechanical alignment of the optical components.

**Figure 5:**
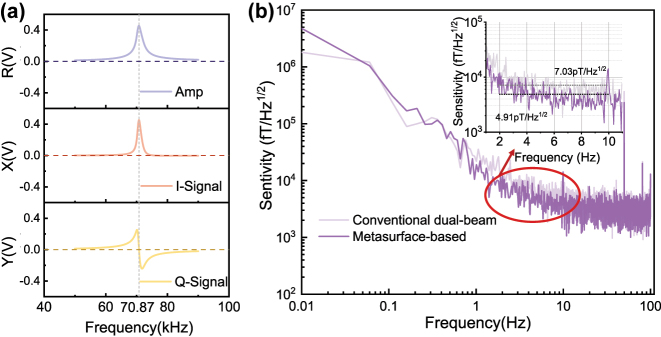
OPM system performance based on polarized metasurface. (a) OPM magnetic resonance signal at 10 μT magnetic field. I-signal: in-phase signal. Q-signal: quadrature-phase signal. (b) Sensitivity of OPM based on polarized metasurface and dual-beam OPM based on conventional optics at 10 μT magnetic field.

In an ensemble of polarized atoms, the application of an RF magnetic field with a frequency matching the Zeeman sub-level splitting is applied, induces magnetic resonance. Under a bias magnetic field of *B*_0_ = 10.14 μT, the metasurface-based OPM demodulated magnetic resonance signals – both in-phase and quadrature components – as shown in Figure 5a. The Larmor precession frequency is given by *ω*_1_ = *γB*_0_, and given that the ^87^Rb gyromagnetic ratio of *γ* = 6.99 Hz/nT, the bias field corresponds to a magnetic resonance center frequency of 70.87 kHz. In OPMs, alkali metal atoms strongly absorb pump and probe beams at resonance frequencies. For the pump beam, proper detuning reduces the coupling strength with atomic resonance, suppresses resonant absorption, and enables more uniform atomic polarization, which benefits sensitivity. For the probe beam, slight detuning can reduce perturbation to atomic populations, minimize unwanted excitations, and adjust the linearity of the magnetic resonance signal, thus improving sensitivity. Since the pump and probe beams share the same laser source, it is essential to detune the laser appropriately near the D_1_ line to minimize the absorption of the probe beam. After multiple rounds of rigorous parameter verification, optimal OPM performance was achieved when the 795 nm laser was detuned by 120–200 GHz. For quantification, the average sensitivity within a specific frequency range is taken as the sensitivity value. Experimental results indicate that the metasurface-based OPM attains a sensitivity of 4.91 pT/Hz^1/2^ in the 2–10 Hz frequency range. Additionally, we performed experiments on a conventional dual-beam OPM with a comparable configuration. As shown in Figure 5b, the metasurface-based dual-beam OPM enhances the sensitivity from 7.03 pT/Hz^1/2^ to 4.91 pT/Hz^1/2^ compared to the conventional system. The optimization process of the magnetometers is described in Supplementary Material S2.

While our metasurface magnetometer does not allow for independent optimization of the detuning between the pump and probe beams as in traditional dual-beam magnetometers, its strength resides in high-precision polarization control. The metasurface used in this work provides stable, high-quality circular polarization under various incident linear polarization azimuth, effectively suppressing noise fluctuations caused by polarization disturbances. Low linear polarization crosstalk also helps reduce spin depolarization. Additionally, the proposed metasurface magnetometer features a simplified structure, a reduced footprint, and less complex optical alignment procedures. The simplified device design reduces potential device-induced noise, which is beneficial for precise polarization and control of thermal atomic ensembles. As a result, the metasurface-based system achieves improved detection sensitivity.

## 3 Conclusions

In summary, we propose and demonstrate a dual-beam atomic magnetometer utilizing a single-frequency laser combined with an LTC metasurface. The polarized metasurface generates a geometric phase through a nanoarray of periodically rotated unit cells. Through careful design and optimization, the metasurface is capable of converting the polarization of incident laser light with arbitrary polarization azimuth, and exhibits an average ellipticity of 44.18° with an axial ratio not exceeding 0.53 dB. By leveraging anomalous refraction, the beam splitting of the converted LCP, RCP and zeroth-order light LP is achieved. While ensuring the spatial separation of the obliquely refracted circularly polarized light from the unconverted light, the zeroth-order LP is used as the probe beam. A dual-beam magnetometer can be constructed using only a single laser source and a polarized metasurface device. The sensitivity of this metasurface OPM in an unshielded environment can reach 5 pT/Hz^1/2^ in the range of 2–10 Hz, surpassing a comparable configuration based on conventional optical components. The system provides enhanced dual-beam magnetometer sensitivity performance, while featuring reduced complexity, compact size and cost efficiency. The metasurface-based OPM in this work not only provides a new solution for OPM performance enhancement, but also facilitates the development of applications such as geological exploration and health monitoring achieving enhanced sensitivity, low power consumption, and high portability.

## Supplementary Material

Supplementary Material Details
