# Supplementary material for: Metasurface-integrated atomic magnetometer using single-frequency dual beams
Source: Nanophotonics. 2025 Sep 4;14(19):3191–9. doi: 10.1515/nanoph-2025-0295 (PMC12455404; doi:10.1515/nanoph-2025-0295)
Supplement: Supplementary file 1 — Supplementary Material Details [file j_nanoph-2025-0295_suppl_001.docx]

Supplementary Materials for

Metasurface-integrated Atomic Magnetometer Using Single-frequency Dual Beams

Ruofan Li ^1,3,4^, Shuo Sun ^1,2,3,4^, Jiahao Zhang ^1,3,4^, and Jin Li ^1,3,4,*^

^1^School of Instrumentation and Optoelectronic Engineering, Beihang University, Beijing 100191, China

^2^College of Optical and Electronic Technology, China Jiliang University, Hangzhou, Zhejiang 310018, China

*^3^National Institute of Extremely-Weak Magnetic Field Infrastructure, Hangzhou, Zhejiang 310051, China*

*email: 11269@buaa.edu.cn

**S1: Performance and Stability Characterization of the Metasurface**

**S1.1 Polarization Purity and Stability of the Zeroth-order Light**

To evaluate the polarization purity and stability of the zeroth-order beam, we systematically characterized its polarization state from three aspects: measurement of polarization purity, polarization retention under different incident conditions, and temporal stability.

To assess the polarization purity, we measured the polarization response of the metasurface under varying incident polarization azimuths, while keeping other experimental parameters constant (detuning: 132.99 GHz, power: 7.08 mW). The response of the zeroth-order light to changes in the incident polarization is shown in the **Fig. S1**. The results show that the transmitted light maintains a high degree of polarization (DOP) with an average value of 98.43% across the full range of incident polarization angles, indicating negligible depolarization. Additionally, the transmitted polarization azimuth exhibits a near-linear relationship with the incident azimuth, with a slope close to 1, suggesting strong polarization-preserving performance. Under different incident polarization azimuths, the deviation between the transmitted and incident polarization angles remains within ±0.6° in most cases. This trend remains consistent even when the incident laser parameters are changed. These results confirm that the metasurface preserves the polarization state of the zeroth-order transmitted light well, which is consistent with theoretical expectations.

**Fig. S1:** Response of zeroth-order light to incident polarization changes.

To evaluate the polarization purity of the zeroth-order transmitted light, we measured the metasurface’s polarization response under different laser detuning conditions. Other experimental parameters were held constant and set to reasonable values: incident linearly polarized light with an ellipticity of –0.64°, azimuth angle of –0.05°, degree of polarization (DOP) of 99.25%, and power of 7.08 mW. The response of the transmitted light to varying detuning conditions is shown in the **Fig. S2**. Experimental results show that across all detuning values, the variations in output polarization angle, azimuth angle, and DOP remain within ±0.07°, ±0.02°, and ±0.05%, respectively, with standard deviations of 0.019, 0.011, and 0.018. Repeating the measurements under different input laser parameters yielded the same trend. These results indicate that the metasurface maintains stable and consistent polarization-preserving performance under different wavelength detuning in the zeroth-order transmission direction.

**Fig. S2:** Response of zeroth-order light to incident laser detuning.

We also measured the polarization response of the metasurface under different incident power conditions. Other experimental parameters were kept constant and set to reasonable values: incident linearly polarized light with an ellipticity of –0.64°, azimuth angle of –0.05°, DOP of 99.25%, and a detuning of 132.99 GHz. The response of the zeroth-order transmitted light to varying incident powers is shown in the **Fig. S3**. The results show that, across all power levels, the deviations in output polarization angle, azimuth angle, and DOP remain within ±0.08°, ±0.17°, and ±1.97%, respectively, with standard deviations of 0.017, 0.043, and 0.53. A slight deviation was observed at an incident power of 0.017 mW due to the low signal intensity, but it does not affect the overall performance. Repeating the measurements with different input laser parameters produced the same trend. These results indicate that the metasurface maintains stable and consistent polarization-preserving performance under varying incident powers in the zeroth-order transmission direction.

**Fig. S3:** Response of zeroth-order light to incident laser power.

We continuously monitored the polarization parameters of the zeroth-order transmitted light for 60 minutes under fixed incident conditions. Other experimental variables were kept constant at reasonable values: polarization azimuth of –0.61°, ellipticity angle of –0.07°, degree of polarization (DOP) of 99.26%, detuning of 132.99 GHz, and incident power of 7.08 mW. The response curves of the polarization state over time are shown in the **Fig. S4**. The results indicate that during the measurement period, the variations in the ellipticity, polarization azimuth, and DOP of the output light remained within ±0.03°, ±0.02°, and ±0.03%, respectively, with standard deviations of 0.0134, 0.0080, and 0.0076. The polarization state remained nearly unchanged. Repeating the measurement under different laser input parameters yielded similar trends. These results confirm that the metasurface exhibits excellent temporal stability of the polarization state in the zeroth-order transmission direction over the measurement period.

**Fig. S4:** Response of zeroth-order light to time.

**S1.2 Polarization Purity and Stability of the converted LCP and RCP**

To ensure high-precision polarization control, we experimentally verified the polarization conversion performance of the metasurface used in this work under varying incident wavelength detuning, power levels, and over time, to assess its stability against system fluctuations. We defined three polarization state metrics to quantify the stability of the metasurface’s polarization conversion:

(1) Ellipticity retention ratio, defined as the ratio of the current measured ellipticity to the initial ellipticity, indicating the stability of the metasurface’s polarization performance.

(2) Azimuth retention ratio, defined as the relative preservation of the polarization principal axis angle, measuring the consistency between the current and initial polarization orientations.

(3) Degree of Polarization (DOP) retention ratio, defined as the ratio of the current DOP to the initial DOP, used to evaluate depolarization trends.

With an incident 795 nm laser power of 4.52 mW, polarization states of RCP and LCP were measured under different wavelength detuning, yielding the curves shown in the **Fig. S5** and **Fig. S6**. The maximum deviations for RCP were 0.43% (ellipticity retention ratio), 2.7% (azimuth retention ratio), and 0.38% (DOP retention ratio); for LCP, the corresponding values were 0.92%, 0.81%, and 0.11%. Similar results were observed at other laser powers, indicating good stability of the metasurface’s polarization conversion across different wavelength detuning.


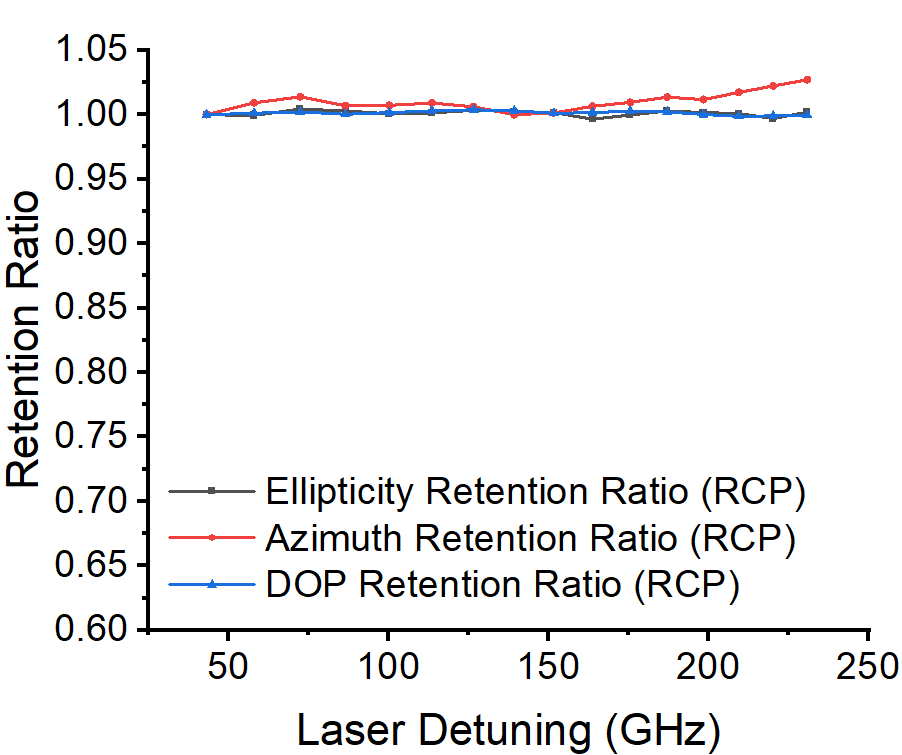


**Fig. S5:** Retention ratios of polarization parameters for RCP under different laser detuning.

**Fig. S6:** Retention ratios of polarization parameters for LCP under different laser detuning.

With the 795 nm laser detuning set at 132.99 GHz, polarization states of RCP and LCP were measured under varying incident laser powers, producing the curves shown in the **Fig. S7** and **Fig. S8**. Due to weaker signals at low powers causing some variation in RCP azimuth angle, the third sampling point was chosen as the reference state. The maximum deviations for RCP were 9.0% (ellipticity retention ratio), 1.1% (azimuth retention), and 8% (DOP preservation); for LCP, the deviations were 5%, 1.8%, and 0.59%, respectively. Similar results were observed at other laser detuning, indicating good stability of the metasurface’s polarization conversion across different incident laser powers.

**Fig. S7:** Retention ratios of polarization parameters for RCP at different incident laser powers.

**Fig. S8:** Retention ratios of polarization parameters for LCP at different incident laser powers.

With the 795 nm laser detuning set at 132.99 GHz and incident power at 4.52 mW, the polarization states of RCP and LCP were measured over sixty minutes. The curves shown in the **Fig. S9** and **Fig. S10** indicate that the maximum deviations for RCP in ellipticity retention, azimuth retention, and DOP preservation were 0.052%, 0.3%, and 0.2%, respectively. For LCP, the corresponding maximum deviations were 0.2%, 0.033%, and 0.02%. Similar results were observed for other laser detuning, powers, and longer measurement durations, demonstrating good temporal stability of the metasurface’s polarization conversion properties.

**Fig.S9:** Retention ratios of polarization parameters for RCP over 60 minutes.

**Fig. S10:** Retention ratios of polarization parameters for LCP over 60 minutes.

The above data and analysis demonstrate that the metasurface’s polarization conversion properties exhibit strong stability against system fluctuations. While high-quality conventional polarization optics can produce purer circular CP light when optimally aligned, this typically requires precise angle adjustments and fixed input polarization axes. In contrast, our metasurface generates CP light from LP input at any incident azimuth without mechanical tuning. This passive robustness simplifies the system and facilitates miniaturization of atomic magnetometers.

To further explain the sensitivity improvement enabled by high-precision polarization control, we start from atomic spin dynamics described by the Bloch equations. These show that spin polarization efficiency depends on the match between the pump beam’s polarization state and atomic transition selection rules. Compared to linear polarization, circularly polarized light more effectively drives single Δm = ±1 transitions, avoiding simultaneous excitation of multiple Zeeman sublevels and enhancing spin polarization directionality and purity. In practice, impurities of linear polarization in circularly polarized pump beam create competing pump vectors that perturb the spin system, causing state mixing and depolarization, reducing steady-state polarization and signal-to-noise ratio.

CP is inherently more robust, especially when polarization purity remains high, as it is less sensitive to small angular or intensity fluctuations, leading to stable signal amplitude and reduced noise. The metasurface-generated LCP light in this work achieves polarization purity comparable to high-precision waveplate systems but without requiring precise mechanical adjustments, maintaining stable CP output under varying input polarization conditions. This stability effectively suppresses noise fluctuations caused by polarization disturbances and, within the Bloch dynamics framework, results in higher steady-state spin polarization and thus improved detection sensitivity.

Moreover, high-precision circular polarization efficiently utilizes pump power by increasing the fraction of useful circularly polarized light that polarizes rubidium atoms, making the effective pump power closer to the incident power of CP light in the cell. Therefore, optimizing pump power in the magnetometer under metasurface-generated high-purity CP pumping effectively optimizes the incident CP power, better supporting targeted magnetometer performance enhancement.

**S2 Optimization Experiments of the Magnetometer**

The metasurface used in this work generates LCP light with polarization purity comparable to that of high-precision waveplate systems. Its low linear polarization impurity helps suppress spin depolarization. Moreover, it provides stable, high-quality circularly polarized output under various input polarization conditions. This stability effectively reduces system noise fluctuations caused by polarization disturbances and leads to higher steady-state spin polarization within the Bloch dynamics framework. The simplified optical setup also reduces potential device-induced noise, which is beneficial for the precise polarization and control of thermal atomic ensembles. As a result, the metasurface-based system achieves improved detection sensitivity. Comparative experiments further demonstrate the advantage of using metasurfaces to generate high-purity circular polarization in enhancing the performance of optically pumped atomic magnetometers (OPMs) under different experimental conditions.

To compare the performance of traditional OPMs and OPM using polarization metasurfaces, we built three types of magnetometers:

(1) Atomic magnetometer using 795 nm pump beam and 780 nm probe beam with conventional optics and dual beams;

(2) Atomic magnetometer using single-frequency 795 nm dual beams with conventional optics;

(3) Metasurface-integrated atomic magnetometer using single-frequency 795 nm dual beams.

The corresponding experimental setups are shown in **Fig. S11**, **Fig. S12**, and **Fig. S13**.


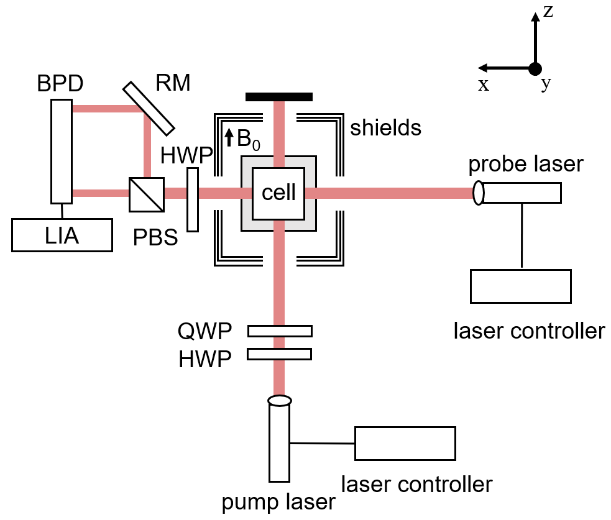


**Fig. S11:** Structural diagram of atomic magnetometer using 795 nm pump beam and 780 nm probe beam with conventional optics and dual beams.


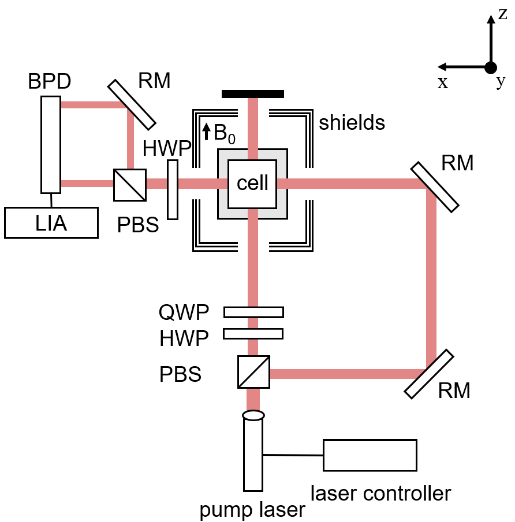


**Fig. S12:** Structural diagram of atomic magnetometer using single-frequency 795 nm dual beams with conventional optics.

**
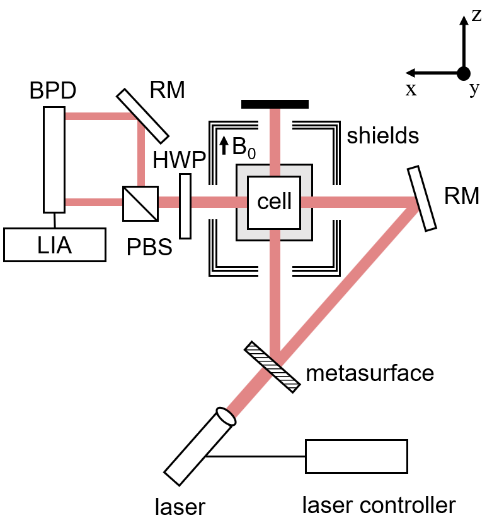
**

**Fig. S13:** Structural diagram of metasurface-integrated atomic magnetometer using single-frequency 795 nm dual beams.

Due to differences in optical paths and energy distribution methods, it is difficult to achieve a controlled single-variable comparison between the three types of magnetometers. Therefore, we adopted the approach of optimizing each magnetometer individually before comparing their performance. For experimental simplicity, we primarily estimate magnetometer performance based on the amplitude and full width at half maximum (FWHM) of the magnetic resonance signal. Under single-variable variation, higher amplitude and narrower FWHM typically indicate better sensitivity. In addition to this indirect evaluation method, we also present sensitivity curves at key operating points as direct data. We take the average sensitivity over the relevant frequency band as the representative sensitivity value. This quantification method allows for a more accurate representation of sensitivity.

**S2.1 Atomic magnetometer using 795 nm pump beam and 780 nm probe beam with conventional optics and dual beams**

(1) We investigated the influence of the RF coil voltage amplitude on the magnetic resonance signal. The parameters other than the RF amplitude were fixed as follows: 795 nm laser power entering the vapor cell was 1.97 mW with a detuning of 145.53 GHz, 780 nm laser power was 3.47 mW with a detuning of 41.58 GHz, cell temperature was 95 °C, and the measured magnetic field was 10.14 μT. The resonance signal behavior under varying RF amplitudes is shown in the **Fig. S14** and **Fig. S15**. When the RF amplitude exceeds 20 mV, the resonance curve exhibits a dip near the peak due to saturation, which complicates analysis. Additionally, increasing RF amplitude beyond this point results in smaller relative improvements in amplitude compared to the broadening of FWHM, which does not benefit sensitivity. We therefore chose 10 mV as the optimal RF amplitude: it produces a clean resonance curve without dips, a relatively high amplitude for clear observation of further optimizations, and a narrow FWHM.

**Fig. S14:** Effect of RF voltage amplitude on the magnetic resonance signal of the atomic magnetometer using 795 nm pump beam and 780 nm probe beam with conventional optics and dual beams.


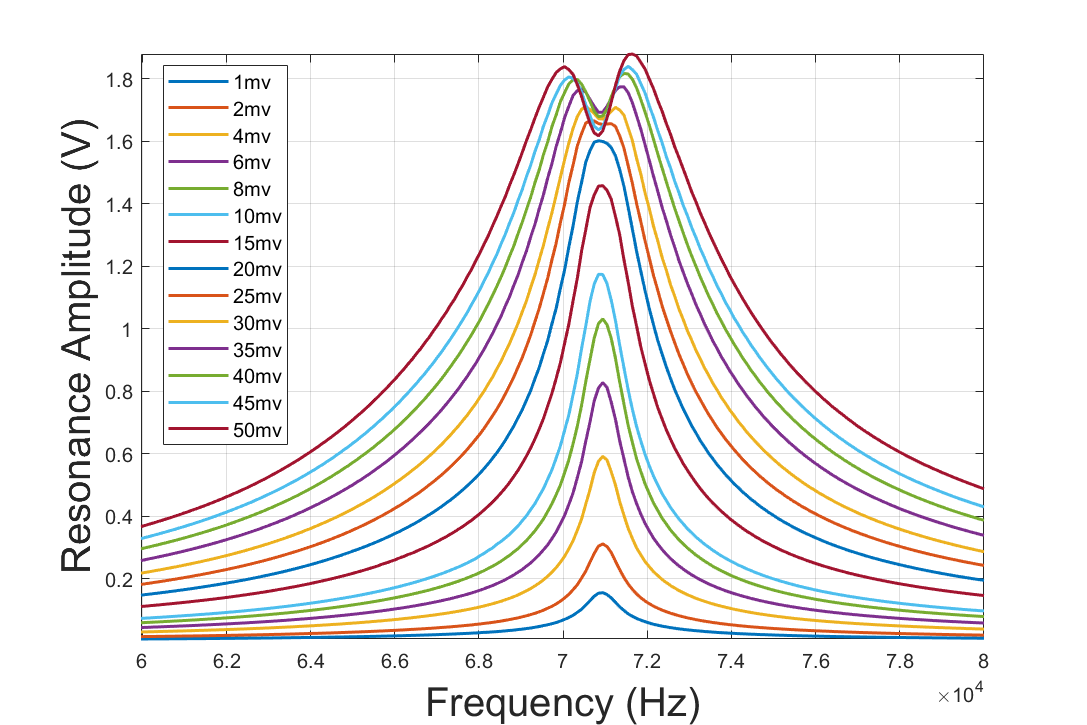


**Fig. S15:** Magnetic resonance curves of the atomic magnetometer using 795 nm pump beam and 780 nm probe beam with conventional optics and dual beams at different RF voltage amplitudes.

(2) We investigated the effect of vapor cell temperature on the magnetic resonance signal. The controlled parameters were as follows: the 795 nm laser power incident on the cell was 1.97 mW with a detuning of 145.53 GHz; the 780 nm laser power was 3.47 mW with a detuning of 41.58 GHz; the RF driving amplitude was 10 mV; and the applied magnetic field was 10.14 μT. The variation of the resonance signal with cell temperature is shown in the **Fig. S16** and **Fig. S17**.

When the temperature is relatively low, the FWHM remains nearly unchanged as temperature increases. Above 85 °C, both the resonance amplitude and FWHM reach relatively high values. Since the magnetometer sensitivity does not show significant changes within the 80–95 °C range, and the amplitude at 95 °C is higher—making it easier to observe signal changes during fine-tuning—we selected 95 °C as the optimal temperature.

**Fig. S16:** Effect of cell temperature on the magnetic resonance signal of the atomic magnetometer using 795 nm pump beam and 780 nm probe beam with conventional optics and dual beams.


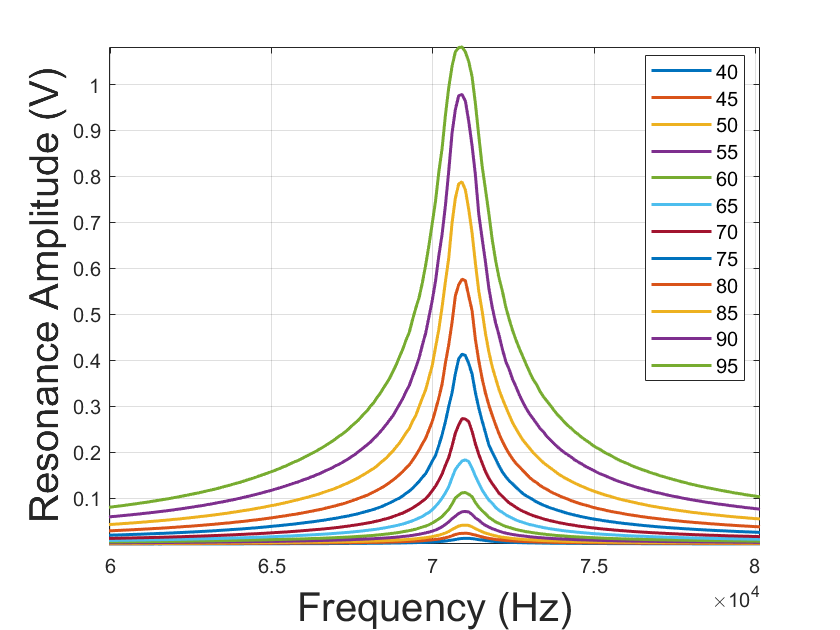


**Fig. S17:** Magnetic resonance curves of the atomic magnetometer using 795 nm pump beam and 780 nm probe beam with conventional optics and dual beams at different cell temperatures.

(3) We investigated the effect of the 795 nm pump laser detuning on the magnetic resonance signal. The controlled parameters were as follows: the 795 nm laser power incident on the vapor cell was 1.97 mW; the 780 nm laser power was 3.47 mW with a detuning of 41.58 GHz; the RF driving amplitude was 10 mV; the vapor cell temperature was 95 °C; and the applied magnetic field was 10.14 μT. The variation of the resonance signal with the 795 nm detuning is shown in the **Fig. S18** and **Fig. S19**.

As shown in the figure, detuning at 139.30 GHz, 145.53 GHz, and 175.59 GHz yield relatively high resonance amplitudes and narrow FWHM, indicating similar sensitivity levels. Among them, the detuning at 145.53 GHz provides both high amplitude and narrow FWHM, along with a more symmetric resonance curve, which is beneficial for improving magnetometer performance. Therefore, we selected 145.53 GHz as the detuning frequency for the 795 nm laser.

**Fig. S18:** Effect of pump detuning on the magnetic resonance signal of the atomic magnetometer using 795 nm pump beam and 780 nm probe beam with conventional optics and dual beams.


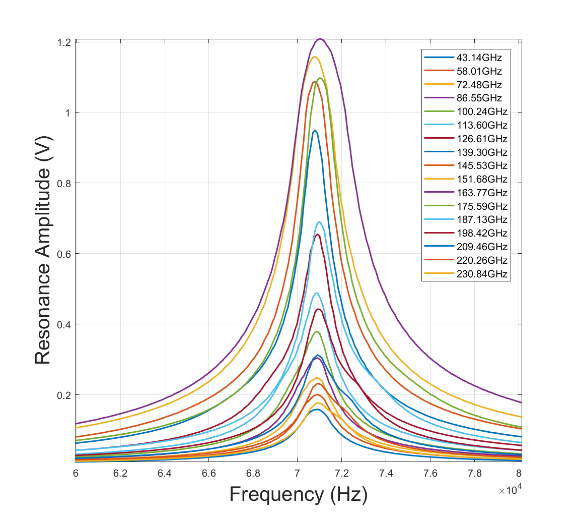


**Fig. S19** Magnetic resonance curves of the atomic magnetometer using 795 nm pump beam and 780 nm probe beam with conventional optics and dual beams at different pump detuning.

(4) We conducted experiments to investigate the effect of the 780 nm probe beam detuning on the magnetic resonance signal. The controlled parameters were as follows: the 795 nm laser power entering the vapor cell was 1.97 mW with a detuning of 145.53 GHz; the 780 nm laser power was 3.47 mW; the RF amplitude was 10 mV; the vapor cell temperature was 95 °C; and the target magnetic field was 10.14 μT. The magnetic resonance signal characteristics of the OPM under different probe beam detuning are shown in the **Fig. S20** and **Fig. S21**.

According to the results, detuning values of 86.35 GHz, 110.38 GHz, and 122.00 GHz yield both high resonance amplitudes and narrow full width at half maximum (FWHM), indicating similar sensitivity. Among them, the 86.35 GHz detuning offers a high resonance amplitude, narrow FWHM, and a symmetric and smooth resonance curve, which are favorable for enhancing magnetometer performance. Therefore, we selected 86.35 GHz as the detuning frequency for the 780 nm laser.

**Fig. S20:** Effect of probe detuning on the magnetic resonance signal of the atomic magnetometer using 795 nm pump beam and 780 nm probe beam with conventional optics and dual beams.


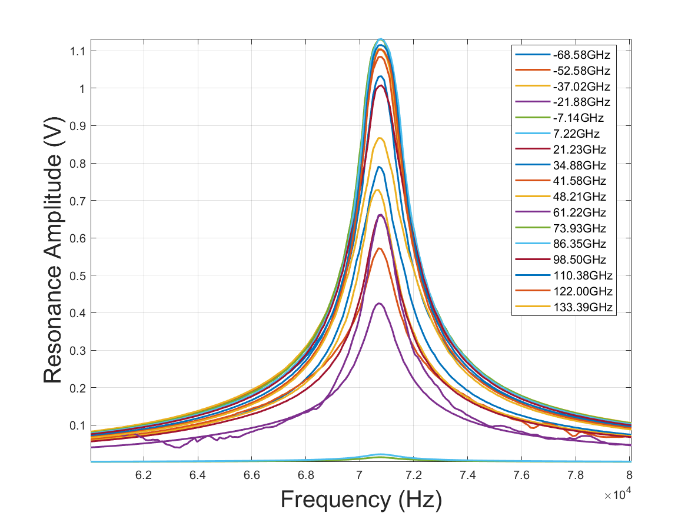


**Fig. S21:** Magnetic resonance curves of the atomic magnetometer using 795 nm pump beam and 780 nm probe beam with conventional optics and dual beams at different probe detuning.

(5) An experiment was conducted to study the effect of 795 nm pump laser power on the magnetic resonance signal. The controlled parameters were as follows: 795 nm laser detuning of 145.53 GHz, 780 nm laser power entering the cell at 3.47 mW with a detuning of 86.35 GHz, RF amplitude of 10 mV, cell temperature of 95°C, and an applied magnetic field of 10.14 μT. The variations in the magnetic resonance signal and the sensitivity curve in the 2–10 Hz frequency range of the OPM with changing 795 nm pump power are shown in the **Fig. S22, Fig. S23** and **Fig. S24**. The best sensitivity performance was observed in the pump power ranges of 1.94–4.38 mW and 6.56–8.17 mW, where the resonance signal showed both higher amplitude and narrower linewidth. At pump powers of 7.72 mW and 4.38 mW, the system reached the first and second optimal sensitivity points, respectively.

These sensitivities were obtained without fine-tuning the probe laser power and thus do not represent the final optimized conditions. Since the pump powers corresponding to the optimal points (7.72 mW and 4.38 mW) are relatively high and the sensitivity improvement is limited, we selected 1.97 mW as the pump power for subsequent probe power optimization. At this point, the system maintains a high resonance amplitude and low linewidth, with significantly reduced power consumption and nearly unchanged sensitivity.

**Fig. S22:** Effect of incident pump power on the magnetic resonance signal of the atomic magnetometer using 795 nm pump beam and 780 nm probe beam with conventional optics and dual beams.


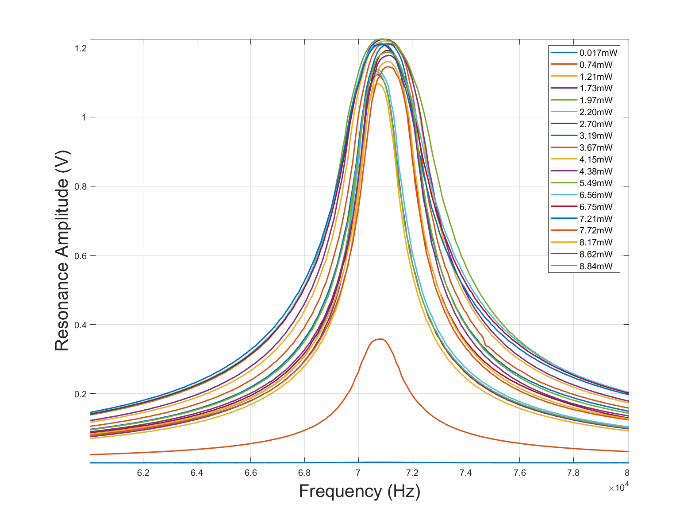


**Fig. S23:** Magnetic resonance curves of the atomic magnetometer using 795 nm pump beam and 780 nm probe beam with conventional optics and dual beams at different incident pump powers.

**Fig. S24:** Sensitivity variation curve of atomic magnetometer using 795 nm pump beam and 780 nm probe beam with conventional optics and dual beams versus incident pump power.

(6) An experiment was conducted to investigate the effect of 780 nm probe laser power on the magnetic resonance signal. The controlled parameters were: 795 nm pump laser power of 1.97 mW with a detuning of 145.53 GHz, 780 nm probe laser detuning of 86.35 GHz, RF amplitude of 10 mV, cell temperature of 95°C, and an applied magnetic field of 10.14 μT. As the 780 nm probe power varied, the magnetometer’s magnetic resonance signal and average sensitivity in the 2–10 Hz frequency range changed accordingly, as shown in the **Fig. S25, Fig. S26** and **Fig. S27**. The sensitivity performed best within the 3.14–3.47 mW probe power range, where the resonance signal exhibited both high amplitude and narrow linewidth. The system achieved optimal sensitivity at a probe power of 3.14 mW.

**Fig. S25:** Effect of incident probe power on the magnetic resonance signal of the atomic magnetometer using 795 nm pump beam and 780 nm probe beam with conventional optics and dual beams.


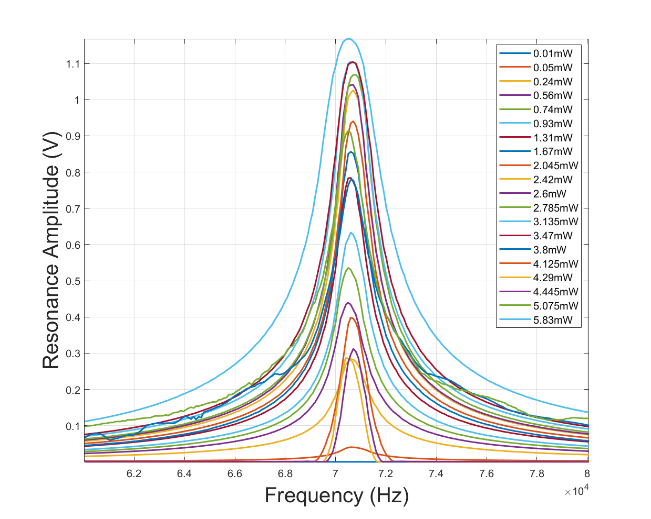


**Fig. S26:** Magnetic resonance curves of the atomic magnetometer using 795 nm pump beam and 780 nm probe beam with conventional optics and dual beams at different incident probe powers.

**Fig. S27:** Sensitivity variation curve of atomic magnetometer using 795 nm pump beam and 780 nm probe beam with conventional optics and dual beams versus incident probe power.

After optimizing six parameters—RF voltage amplitude, cell temperature, 795 nm pump laser detuning, 780 nm probe laser detuning, 795 nm pump power, and 780 nm probe power—the sensitivity curve of the atomic magnetometer using 795 nm pump beam and 780 nm probe beam with conventional optics and dual beams is shown in the **Fig. S28**. The magnetometer achieved sensitivities of 7.03 pT/Hz^1/2^ in the 2–10 Hz band and 4.14 pT/Hz^1/2^ pT/Hz^1/2^ in the 10–40 Hz band.

**Fig. S28:** Sensitivity of atomic magnetometer using 795 nm pump beam and 780 nm probe beam with conventional optics and dual beams.

(7) The system was set to optimal parameters: RF voltage amplitude of 10 mV, cell temperature of 95 °C, 795 nm pump laser detuning of 145.53 GHz, and 780 nm probe laser detuning of 86.35 GHz. First, the optical power loss under different pump powers was measured (with the 780 nm laser fixed at 3.14 mW). Then, the optical power loss under different probe powers was measured (with the 795 nm laser fixed at 1.97 mW). The corresponding optical power loss curves are shown in the **Fig. S29** and **Fig. S30**.

Under optimal detuning for both the 795 nm and 780 nm lasers, the power loss in the cell was measured to be 0.88 mW for the pump beam and 1.53 mW for the probe beam.

**Fig. S29:** Effect of incident pump power on optical power loss in the vapor cell.

**Fig. S30:** Effect of incident probe power on optical power loss in the vapor cell.

**S2.2 Atomic magnetometer using single-frequency 795 nm dual beams with conventional optics**

(1) An experiment was conducted to study the effect of RF coil voltage amplitude on the magnetic resonance signal. Parameters other than the RF amplitude were kept constant as follows: 795 nm pump laser power entering the cell at 0.76 mW, probe laser power at 0.71 mW, laser detuning of 145.53 GHz, cell temperature at 95°C, and an applied magnetic field of 10.14 μT. To facilitate comparison with the metasurface-based atomic magnetometer using single-frequency dual beams, the pump and probe powers were set nearly equal. As the RF amplitude varied, the magnetic resonance signal characteristics changed as shown in the **Fig. S31,** and **Fig. S32**. When the RF amplitude exceeded 25 mV, the resonance peak saturated and developed a dip, complicating analysis. Moreover, at higher RF amplitudes, the increase in resonance amplitude was smaller than the increase in linewidth, resulting in no performance improvement over lower RF values. Balancing these factors, we selected an RF amplitude of 10 mV, which yielded a normal resonance curve without dips, with relatively high amplitude for easier observation during subsequent optimization, and a narrow linewidth.

**Fig. S31:** Effect of RF voltage amplitude on the magnetic resonance signal of the atomic magnetometer using single-frequency 795 nm dual beams with conventional optics.


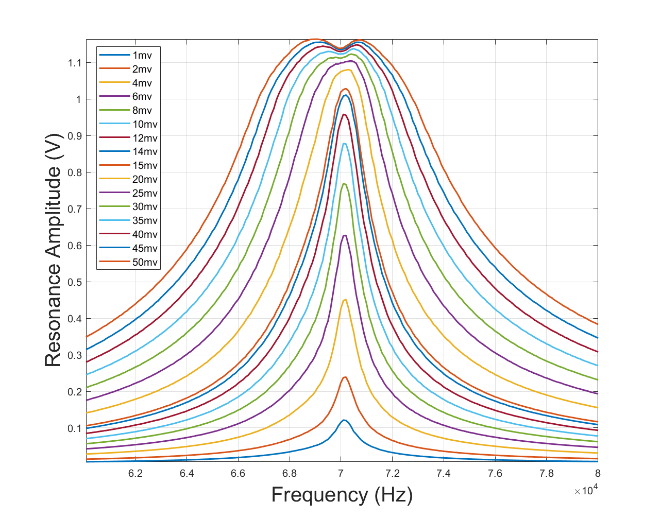


**Fig. S32:** Magnetic resonance curves of the atomic magnetometer using single-frequency 795 nm dual beams with conventional optics at different RF voltage amplitudes.

(2) An experiment was conducted to investigate the effect of cell temperature on the magnetic resonance signal. Controlled parameters were: 795 nm pump power of 0.76 mW, probe power of 0.71 mW, laser detuning of 145.53 GHz, RF amplitude of 10 mV, and an applied magnetic field of 10.14 μT. The magnetic resonance signal varied with cell temperature as shown in the **Fig. S33** and **Fig. S34**.

At lower temperatures, the resonance linewidth was broad and the amplitude was low, resulting in poor signal quality and difficulty in phase tracking. Above 90°C, the resonance amplitude increased while the linewidth decreased, improving signal quality, with a favorable amplitude-to-linewidth ratio. Temperatures between 90°C and 100°C generally had minimal impact on magnetometer performance, mainly affecting the optimal parameter settings. At 95°C, the resonance amplitude was sufficiently high for easier observation during fine parameter tuning and consistent with previous magnetometer conditions for comparison. Considering these factors, 95°C was selected as the operating cell temperature.

**Fig. S33:** Effect of cell temperature on the magnetic resonance signal of the atomic magnetometer using single-frequency 795 nm dual beams with conventional optics.


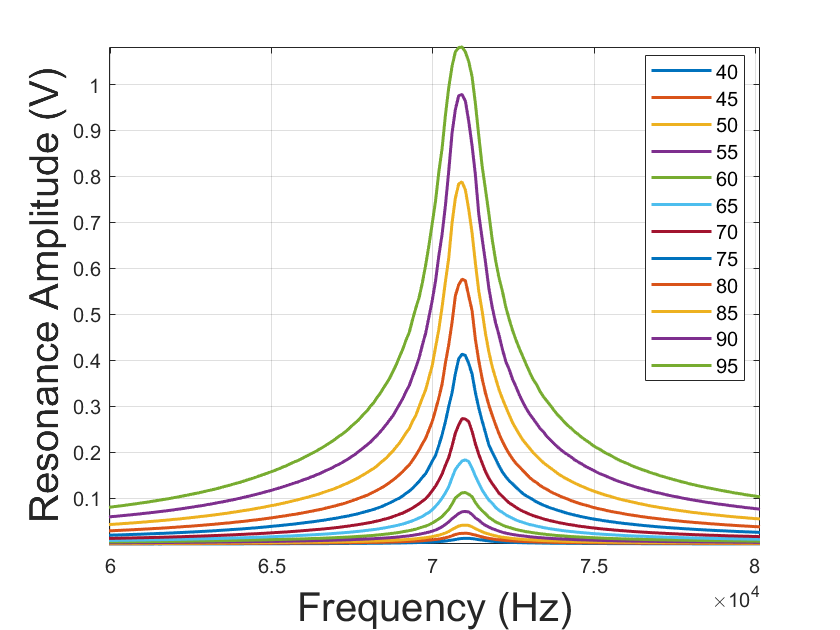


**Fig. S34:** Magnetic resonance curves of the atomic magnetometer using single-frequency 795 nm dual beams with conventional optics at different cell temperatures.

(3) An experiment was performed to study the effect of 795 nm laser detuning on the magnetic resonance signal. The controlled parameters were: 795 nm pump power of 0.76 mW, probe power of 0.71 mW, RF amplitude of 10 mV, and an applied magnetic field of 10.14 μT. The magnetic resonance signal varied with detuning as shown in the **Fig. S35** and **Fig. S36**.

Analysis of the data indicates that at a detuning of 139.30 GHz, the resonance exhibits a high amplitude, narrow linewidth, and a symmetric, smooth lineshape, resulting in a large amplitude-to-linewidth ratio favorable for magnetometer performance. Therefore, 139.30 GHz was selected as the 795 nm laser detuning frequency.

**Fig. S35:** Effect of pump detuning on the magnetic resonance signal of the atomic magnetometer using single-frequency 795 nm dual beams with conventional optics.


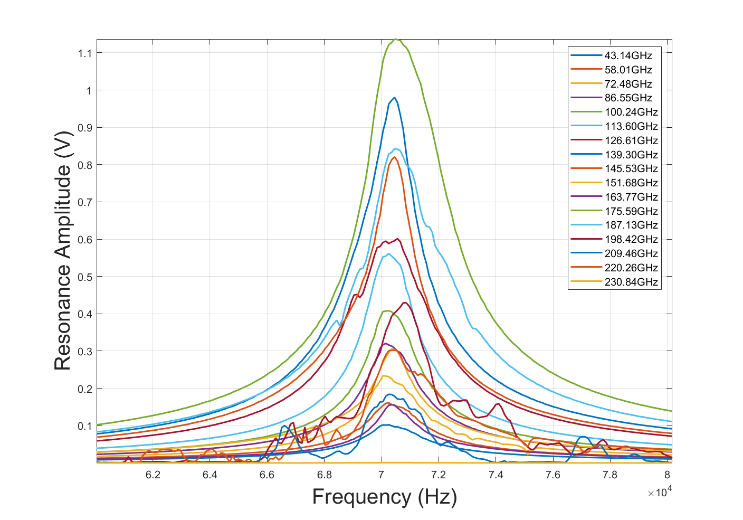


**Fig. S36:** Magnetic resonance curves of the atomic magnetometer using single-frequency 795 nm dual beams with conventional optics at different pump detuning.

(4) An experiment was conducted to investigate the effect of 795 nm laser power on the magnetic resonance signal. Controlled parameters were: 795 nm laser detuning of 145.53 GHz, RF amplitude of 10 mV, and an applied magnetic field of 10.14 μT. The magnetic resonance signal varied with the 795 nm laser power as shown in the **Fig. S37** and **Fig. S38**.

Analysis shows that at a pump power of 1.11 mW, the resonance signal exhibits high amplitude, narrow linewidth, and a symmetric, smooth lineshape, with a large amplitude-to-linewidth ratio favorable for magnetometer performance. However, the signal-to-noise ratio at this point exceeded the magnetometer’s tracking range, requiring an alternative optimal point. At 0.57 mW pump power, the resonance performance remains good and allows stable phase tracking. Therefore, 0.57 mW was selected as the 795 nm laser power.

**Fig. S37:** Effect of incident pump power on the magnetic resonance signal of the atomic magnetometer using single-frequency 795 nm dual beams with conventional optics.


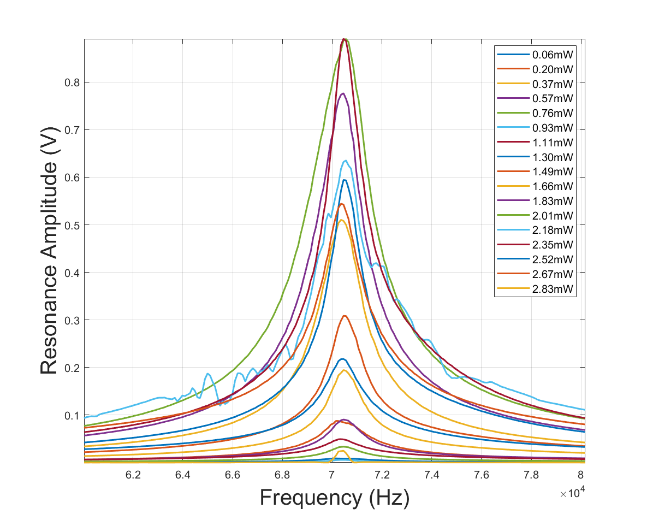


**Fig. S38:** Magnetic resonance curves of the atomic magnetometer using single-frequency 795 nm dual beams with conventional optics at different incident pump powers.

After optimizing four parameters—RF voltage amplitude, cell temperature, 795 nm laser detuning, and 795 nm laser power—the sensitivity curve of the atomic magnetometer using single-frequency 795 nm dual beams with conventional optics is shown in the **Fig. S39**. The magnetometer achieved sensitivities of 9.06 pT/Hz^1/2^ in the 2–10 Hz band and 5.04 pT/Hz^1/2^ in the 10–40 Hz band.

**Fig. S39:** Sensitivity of atomic magnetometer using single-frequency 795 nm dual beams with conventional optics.

(5) The system was set to optimal parameters: RF voltage amplitude of 10 mV, cell temperature of 95 °C, and 795 nm laser detuning of 145.53 GHz. The optical power loss of the pump and probe beams was measured under varying input laser powers, and the corresponding power loss curves are shown in the **Fig. S40** and **Fig. S41**.

Under optimal conditions for the 795 nm laser, the optical power loss in the cell was measured to be 0.328 mW for the pump beam and 0.256 mW for the probe beam, resulting in a total power loss of 0.584 mW.

**Fig. S40:** Effect of incident pump power on optical power loss in the vapor cell.

**Fig. S41:** Effect of incident probe power on optical power loss in the vapor cell.

**S2.3 Metasurface-integrated atomic magnetometer using single-frequency 795 nm dual beams**

(1) An experiment was conducted to study the effect of RF coil voltage amplitude on the magnetic resonance signal. Parameters other than the RF amplitude were fixed as follows: 795 nm pump power of 1.99 mW, laser detuning of 145.53 GHz, cell temperature of 95°C, and an applied magnetic field of 10.14 μT. The magnetic resonance signal varied with RF amplitude as shown in the **Fig. S42, Fig. S43** and **Fig. S44**.

When the RF amplitude exceeded 12 mV, the resonance peak saturated and developed a dip, which complicated analysis. Additionally, at higher RF amplitudes, the increase in resonance amplitude was smaller than the increase in linewidth, resulting in no performance improvement over lower RF values. Balancing these factors, we selected 10 mV, where the resonance curve showed no dip, had a relatively high amplitude for easier observation during further optimization, and a narrow linewidth.

**Fig. S42:** Effect of RF voltage amplitude on the magnetic resonance signal of the metasurface-integrated atomic magnetometer using single-frequency 795 nm dual beams.


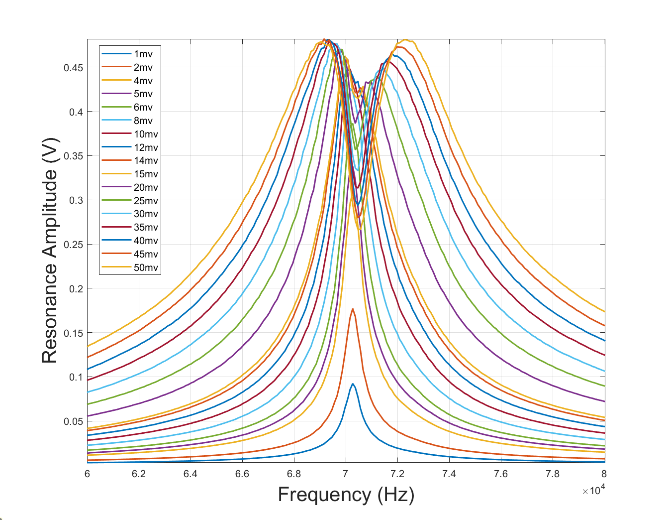


**Fig. S43:** Magnetic resonance curves of the metasurface-integrated atomic magnetometer using single-frequency 795 nm dual beams.


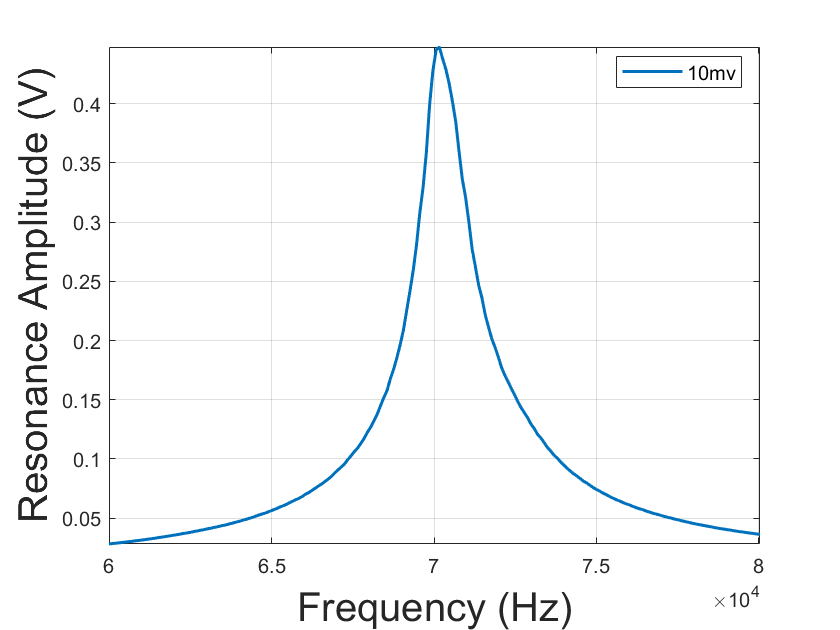


**Fig. S44:** Magnetic resonance curves of the metasurface-integrated atomic magnetometer using single-frequency 795 nm dual beams at an RF voltage amplitude of 10 mV.

(2) An experiment was conducted to investigate the effect of cell temperature on the magnetic resonance signal. Controlled parameters were: 795 nm pump power of 1.99 mW, laser detuning of 145.53 GHz, RF amplitude of 10 mV, and an applied magnetic field of 10.14 μT. The magnetic resonance signal varied with cell temperature as shown in the **Fig. S45** and **Fig. S46**.

At lower temperatures, the resonance linewidth was broad and the amplitude was low, resulting in poor signal quality and difficulty in phase tracking. Above 90°C, the resonance amplitude increased and the linewidth decreased, improving the amplitude-to-linewidth ratio. Temperatures between 90°C and 100°C generally have minimal impact on magnetometer performance, mainly affecting optimal parameter values. At 95°C, the resonance amplitude was high enough for easier observation during fine tuning, despite a slight dip in the curve. This temperature also matches previous magnetometer conditions, facilitating comparison. Considering these factors, 95°C was selected as the operating temperature.

**Fig. S45:** Effect of cell temperature on the magnetic resonance signal of the metasurface-integrated atomic magnetometer using single-frequency 795 nm dual beams.


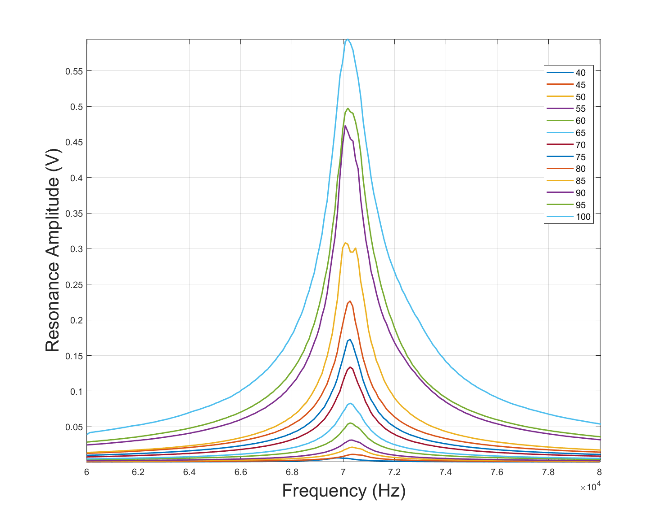


**Fig. S46:** Magnetic resonance curves of the metasurface-integrated atomic magnetometer using single-frequency 795 nm dual beams.

(3) An experiment was conducted to study the effect of 795 nm laser detuning on the magnetic resonance signal. Controlled parameters were: 795 nm pump power of 1.99 mW, RF amplitude of 10 mV, and an applied magnetic field of 10.14 μT. The magnetic resonance signal characteristics and sensitivity curve in the 2–10 Hz range varied with the 795 nm laser detuning, as shown in the **Fig. S47, Fig. S48** and **Fig. S49**.

At 132.991 GHz detuning, the resonance signal had an amplitude of 334.94 mV and a linewidth of 2254 Hz, ranking as the fifth highest peak. This resonance exhibited relatively high amplitude, narrow linewidth, and a smooth lineshape without significant dips or wrinkles, favorable for sensitivity improvement.

On the lower detuning side (43.14–113.60 GHz), the magnetometer failed to track the output phase, preventing system locking. Between 113.56–132.99 GHz, sensitivity decreased with detuning, then increased in the 132.99–230.84 GHz range. Under these conditions, 132.99 GHz detuning yielded the minimum sensitivity, with adjacent regions showing small, similar fluctuations.

Since the pump beam is used for spin polarization and the probe beam for state detection, they have different detuning requirements. Small pump detuning yields high polarization but causes stronger absorption saturation and thermal effects. Large detuning reduces absorption and polarization efficiency, requiring controlled detuning values. Larger probe detuning minimizes disturbance to atomic populations and reduces unwanted excitation.

Based on Bloch dynamics, laser detuning should be optimized to balance pumping efficiency and detection sensitivity for overall system performance. Using the same laser source for both pump and probe beams necessitates precise detuning adjustment. The experiment also shows that laser detuning affects magnetometer stability, phase-tracking ability, and sensitivity.

**Fig. S47:** Effect of pump detuning on the magnetic resonance signal of the metasurface-integrated atomic magnetometer using single-frequency 795 nm dual beams.


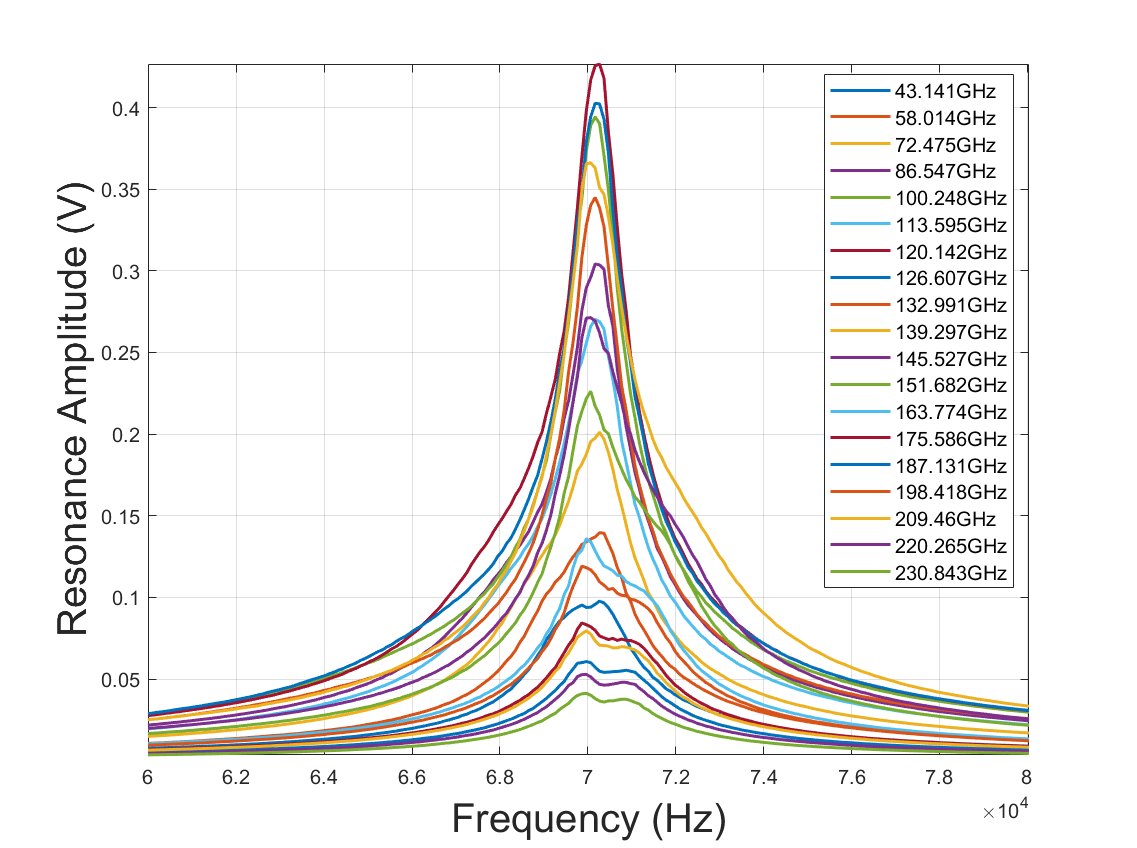


**Fig. S48:** Magnetic resonance curves of the metasurface-integrated atomic magnetometer using single-frequency 795 nm dual beams at different pump detuning.

**Fig. S49:** Sensitivity variation curve of metasurface-integrated atomic magnetometer using single-frequency 795 nm dual beams versus incident pump power.

(4) An experiment was conducted to investigate the effect of 795 nm laser power on the magnetic resonance signal. Controlled parameters were: 795 nm laser detuning of 132.99 GHz, RF amplitude of 10 mV, and an applied magnetic field of 10.14 μT. The magnetic resonance signal characteristics and average sensitivity in the 2–10 Hz range varied with the 795 nm laser power, as shown in the **Fig. S50, Fig. S51** and **Fig. S52**.

Due to the use of a single-frequency laser for both pumping and probing, stable phase-locked output can only be maintained within specific pump power ranges. Outside these ranges, reduced pumping efficiency from spectral overlap or excessive probe absorption prevents effective optical polarization and signal modulation, causing loss of lock.

Stable phase tracking was achieved in the pump power ranges of 0.81–1.22 mW and 1.84–3.53 mW. Among these, the magnetometer showed the best sensitivity with high resonance amplitude and narrow linewidth in the 1.99–2.83 mW range. The optimal sensitivity occurred at a pump power of 2.47 mW.

**Fig. S50:** Effect of pump detuning on the magnetic resonance signal of the metasurface-integrated atomic magnetometer using single-frequency 795 nm dual beams.


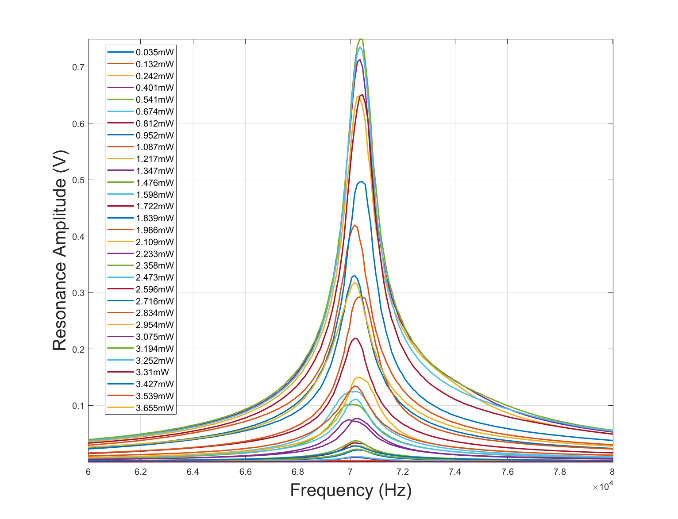


**Fig. S51:** Magnetic resonance curves of the metasurface-integrated atomic magnetometer using single-frequency 795 nm dual beams at different pump detuning.

**Fig. S52:** Sensitivity variation curve of metasurface-integrated atomic magnetometer using single-frequency 795 nm dual beams versus incident pump power.

After optimizing four parameters—RF voltage amplitude, cell temperature, 795 nm laser detuning, and 795 nm laser power—the sensitivity curve of the metasurface-integrated atomic magnetometer using single-frequency 795 nm dual beams is shown in the **Fig. S53**. The magnetometer achieved sensitivities of 4.91 pT/Hz^1/2^ in the 2–10 Hz band and 3.21 pT/Hz^1/2^ in the 10–40 Hz band.

**Fig. S53:** Sensitivity of metasurface-integrated atomic magnetometer using single-frequency 795 nm dual beams.

(5) The system was configured with optimal parameters: RF voltage amplitude of 10 mV, cell temperature of 95 °C, and 795 nm laser detuning of 132.99 GHz. The optical power loss of the pump and probe beams was measured under varying incident laser powers, and the corresponding power loss curves are shown in the **Fig. S54** and **Fig. S55**.

Under optimal conditions for the 795 nm laser, the measured optical power loss in the cell was 1.23 mW for the pump beam and 0.79 mW for the probe beam, resulting in a total power loss of 2.02 mW.

It can be seen that the total optical power consumed by the metasurface-based atomic magnetometer using a single-frequency dual-beam configuration is on the same order of magnitude as that of the conventional magnetometer using a dual-frequency dual-beam configuration, and is slightly lower. Since the total energy absorbed by the high-temperature rubidium vapor cell is lower than that in the conventional design, the associated thermal effects are also not greater.

In summary, the polarization conversion by the metasurface does not lead to increased power consumption or thermal effects in the single-frequency dual-beam atomic magnetometer based on a polarization metasurface.

**Fig. S54:** Effect of incident pump power on optical power loss in the vapor cell.

**Fig. S55:** Effect of incident probe power on optical power loss in the vapor cell.

After careful optimization, the atomic magnetometer using 795 nm pump beam and 780 nm probe beam with conventional optics and dual beams achieved sensitivities of 7.03 pT/Hz^1/2^ and 4.14 pT/Hz^1/2^ in the 2–10 Hz and 10–40 Hz bands, respectively. The atomic magnetometer using single-frequency 795 nm dual beams with conventional optics reached sensitivities of 9.06 pT/Hz^1/2^ and 5.04 pT/Hz^1/2^ in the same bands. The metasurface-integrated atomic magnetometer using single-frequency 795 nm dual beams achieved improved sensitivities of 4.91 pT/Hz^1/2^ and 3.21 pT/Hz^1/2^, showing comparable but relatively better performance than the other two systems.
